# Magnesium dietary intake and physical activity in Type 2 diabetes by gender in White, African‐American and Mexican American: NHANES 2011‐2014

**DOI:** 10.1002/edm2.203

**Published:** 2020-11-20

**Authors:** You Wu, Susmita Datta, Bert B. Little, Maiying Kong

**Affiliations:** ^1^ Global Biostatistics Science Center for Design and Analysis Amgen Inc. Thousand Oaks CA USA; ^2^ Department of Bioinformatics and Biostatistics School of Public Health and Information Sciences University of Louisville Louisville KY USA; ^3^ Department of Biostatistics, College of Public Health & Health Professions and College of Medicine University of Florida 2004 Mowry Rd Gainesville United States FL 32611‐7450 USA; ^4^ Department of Health Management and Systems Sciences School of Public Health and Information Sciences University of Louisville Louisville KY USA

**Keywords:** magnesium intake, NHANES, path analysis, physical activity, T2DM

## Abstract

**Aims:**

To analyse the causal relationships of nutrition intake and physical activity on haemoglobin A1c (HbA1C) in patients diagnosed with type 2 diabetes mellitus (T2DM) stratified by gender and ethnicity.

**Materials and Methods:**

An historical cohort of patients with diagnosed T2DM (n = 2831) was extracted from the National Health and Nutrition Examination Survey (NHANES) 2011‐2014 public database, including but not limited to, measurements of physical activity, nutrition, body mass index (BMI) and HbA1c. Multivariate analyses and path analyses were employed to estimate the regression coefficients and path coefficients (*ρ*) of causal path models of physical activity and nutrition intake on HbA1c stratified by gender and three ethnicity groups (ie non‐Hispanic white, non‐Hispanic black and Mexican American).

**Results:**

A significant causal path from increased physical activity to increased magnesium (Mg) intake to decreased HbA1c was found. In addition, increased physical activity significantly decreased BMI, which further decreased HbA1c. These results varied by gender and ethnicity but were directionally consistent. Physical activity decreased HbA1c through BMI for males and through Mg intake for females. Mexican American decreased HbA1c through Mg intake, while non‐Hispanic black had an increased HbA1c due to its ethnicity and through increased BMI.

**Conclusions:**

The beneficial effects of physical activity on decreased HbA1c were mediated through the increased Mg intake and decreased BMI. This aligned with recent investigations of the inverse causal association of Mg intake with insulin resistance and with decreased inflammation.

## Graphical abstract

We applied causal path analyses to NHANES 2011‐2014 dataset to analyse the causal relationships of nutrition intake and physical activity on HbA1C in patients diagnosed with type 2 diabetes mellitus stratified by gender and ethnicity. We found that the beneficial effects of physical activity on decreased HbA1c were mediated through the increased Mg intake and decreased BMI across different gender and ethnicities.

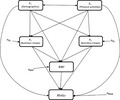

## 1 INTRODUCTION

Type 2 diabetes mellitus (T2DM) is a major public health problem worldwide and is one of the major causes of mortality in the United States (US) outstripping cancer, HIV/AIDS, and cardiovascular diseases. In addition, negative economic and psychosocial outcomes are associated with T2DM.^1^, ^2^, ^3^ The complications from diabetes affect several organ systems and increase the risk of premature death.^4^ T2DM also results in an increased economic burden on the individual and the healthcare system.^3^ Globally, it is estimated that 422 million adults had T2DM in 2014, which was 12% of the adult population.^5^ In the USA, diabetes prevalence rose sharply over the past three decades. Diagnosed T2DM accounted for 3.3% of the US population in 1995, rose to 5.6% in 2005 and was 12.1% in 2015, not including undiagnosed diabetes.^6^ Variation in prevalence of diabetes exists between ethnic groups due to hereditary and environmental factors. The prevalence of T2DM is higher in non‐Hispanic blacks (19.1%) and Mexican Americans (14.5%) compared with non‐Hispanic whites (10.1%).^1^ ^,^ ^6^

Of the two types of diabetes, T2DM comprises approximately 90% to 95%, and Type‐1 diabetes ranges from 5% to 10%.^7^ Type 1 diabetes has a different aetiology from T2DM and is characterized by the total inability of the pancreas to produce insulin, while type 2 diabetes is caused by a combination of genetic and environmental factors related to impaired insulin secretion, insulin resistance, obesity, overeating, lack of exercise, stress, and ageing.^8^ Management of T2DM includes modified nutritional intake, physical activity, and medication as the three key counteracting strategies.^9^ Associations of these factors with haemoglobin A1c (HbA1C) in T2DM have been investigated using regression models^9^, ^10^, ^11^, ^12^, ^13^ and indicated that (a) lifestyle interventions (diet and physical exercise) reduce incidence of T2DM, (b) physical exercise can manipulate blood magnesium levels, particularly in patients with T2DM,and (c) obesity is one of the most important modifiable risk factors for the prevention of type 2 diabetes. The causal relationships of nutrition intake and physical activity on HbA1C in patients diagnosed with T2DM have not been studied using the causal path analysis.^14^, ^15^, ^16^ Path analysis is an extension of multiple regression and a special case of causal structural equation models. Path analysis and causal inference procedures are based on ideas developed originally in biology and economics^14^, ^15^ for cross‐sectional data, where parent‐offspring regressions were used at a single point in time. Path analysis uses a system of structural equations and allows analysis of the relationship between dependent variables as well as between independent variables and dependent variables in a complex system analysis.^17^ Path analysis is very powerful for examining complex models and for studying the causal relationship and mediation effects. Although there are several randomized clinical trials (RCTs) showing that increased physical activity and nutrition (eg Mg supplement) can improve HbA1c in T2D patients^18^, ^19^, these studies are specific for certain populations. The current study is based on NHANES data, and the results could be applicable to the USA general population. In the present investigation, we apply path^14^, ^15^, ^16^ to examine these causal relationships using the National Health and Nutrition Examination Survey (NHANES) 2011‐2014 dataset. The current study's goal is to provide insights into possible mechanisms of how dietary intake of specific nutrients and physical activity affect T2DM management.

## 2 MATERIAL AND METHOD

### 2.1 Data and materials

The NHANES datasets are a publicly available national survey conducted by the National Center for Health Statistics of the US Centers for Disease Control and Prevention. Different NHANES cycles assess various subsets of special studies (eg nutritional intake). The NHANES (2011‐2014) database includes demographic information, physical activity, dietary components, as well as laboratory measurements for HbA1C. NHANES 2011‐2014 datasets analysed in this study contain nutrition intake and the health status for a representative sample in the US population.

NHANES 2011‐2014 datasets included 65 nutritional intake variables (from the dietary dataset) to measure dietary intake for each individual. Subjects 20 to 79 years old were included in the present study. We used two dummy variables for three ethnic groups: non‐Hispanic black, Mexican American and non‐Hispanic white. Listwise deletion of cases with missing values was employed. All the subjects included had a complete record for demographic characteristics (ie age, gender, race and ethnicity), physical activity, BMI, HbA1c and all 65 nutritional intake measures. We only included the subjects who were taking oral hypoglycemic agents (ie biguanides, sulphonylureas and thiazolidinedione‐TZDs). Insulin‐dependent subjects were excluded. Thus, we obtained a cohort of patients with diagnosed T2DM who were taking only oral hypoglycemic medications. The flowchart of the study cohort was presented in Figure S1 in the online supplementary material.

### 2.2 Variables used in the analyses

The distribution of the diet and physical activity in the US adults with T2DM based on NHANES III has been reported^13^ ^,^ but NHANES dietary data have not been used in a causal path model to analyse the effect of physical activity and nutrition intake on HbA1c. In the present study, path analysis^14^, ^15^, ^16^ was used to analyse a system of causal structural models to predict HbA1c using the data on demographics, dietary intake, BMI and physical activity. Causal analysis of nutrition intake, physical activity and BMI on T2DM, across three major US ethnic groups (ie non‐Hispanic white, non‐Hispanic black and Mexican American) may provide insight into medical management of T2DM.

The variable ‘physical activity’ was defined using the NHANES physical activity questionnaire data on weekly work activities and recreational activities. Physical activity in the analysis was a dummy variable where ‘1’ indicates subjects who had either vigorous activities or moderate activities, and ‘0’ indicates no physical inactivity.

Education was scaled as an ordinal Likert five level score: (a) less than 9th grade, (b) 9‐11th grade (including 12th grade with no diploma), (c) high school graduate/general educational development or equivalent, (d) some college or associate degree and (e) college graduate or above.

Socio‐economics status (SES) is measured by a three‐level family monthly poverty level index: less than 1.30, between 1.30 and 1.85, and greater than 1.85. The monthly poverty level index is defined as the ratio of monthly family income to family size according to the Department of Health and Human Services poverty guidelines.^20^ SES is treated as a numerical variable taking scores from 1 to 3 with a higher score indicating a higher SES. Mean and standard deviation (SD) are reported for each variable (see Table 1).

**Table 1:** Descriptive statistics for the variables included in the path analyses for the entire study cohort, stratified by gender and ethnicity

Variables	Non‐Hispanic White					Non‐Hispanic Black					Mexican American				
	Male (n = 750)		Female (n = 822)			Male (n = 360)		Female (n = 454)			Male (n = 229)		Female (n = 216)		
	Mean	SD	Mean	SD	*P*‐value	Mean	SD	Mean	SD	*P*‐value	Mean	SD	Mean	SD	*P*‐value
HbA1c	5.6	0.9	5.6	0.9	.521	6.1	1.6	5.9	1.1	.054	5.8	1.2	5.8	1.2	.824
BMI	29	6.3	29.9	8.1	.023	29.4	8.1	32.6	8.7	<.001	29.3	5.3	30.9	6.6	.006
Age	48.4	16.4	49.1	16.5	.396	50.4	16.1	46.8	15.7	.001	45.9	16.0	45.2	14.7	.608
Education	3.7	1.1	3.8	1.1	.013	3.4	1.1	3.7	1.0	<.001	2.6	1.3	2.7	1.4	.346
SES	2.3	1.1	2.3	1.2	.256	2.2	1.2	2.1	1.3	.143	1.9	1.3	1.9	1.3	.996
Protein (gm)	92	44.4	68.3	31.7	<.001	88.8	46.0	69.4	38.8	<.001	104.3	52.9	75.6	37.5	<.001
Carbohydrate (gm)	277.2	130.3	214.9	100.5	<.001	247.1	123.4	220.1	114.2	.001	299.9	144.6	230.1	101.1	<.001
Total sugars (gm)	123.5	83.6	97.5	60.9	<.001	107.2	70.9	102.5	67.9	.336	118.6	67.9	96.6	58.4	<.001
Total fat (gm)	88.3	45.3	66.8	36.7	<.001	80.9	46.6	69.3	40.6	<.001	90.1	52.4	66.4	37.5	<.001
Magnesium (mg)	326.1	168.7	265	125	<.001	288.7	159.5	247.4	128.9	<.001	367.1	196.7	293.4	131.8	<.001
Moisture (gm)	3122	1524	2756	1261	<.001	2552	1335	2239	1182	<.001	3100	1668	2610	1152	<.001
SFA 4:0 (Butanoic) (gm)	0.6	0.6	0.5	0.5	<.001	0.4	0.5	0.4	0.4	.603	0.5	0.6	0.4	0.4	.009
	**Count**	**%**	**Count**	**%**	***P*‐value**	**Count**	**%**	**Count**	**%**	***P*‐value**	**Count**	**%**	**Count**	**%**	***P*‐value**
Physical Activity	573	76.4%	572	69.6%	.003	264	73.3%	272	59.9%	<.001	162	70.7%	135	62.5%	.070

Note

Mean and standard deviation (SD) are reported for the continuous and ordinal variables, and the frequency and percentage (%) are reported for categorical variable. *P*‐values for two group comparison are calculated using two samples t test for continuous variables and Fisher's exact test for categorical variables.

### 2.3 Statistical analysis

**Figure 1:**
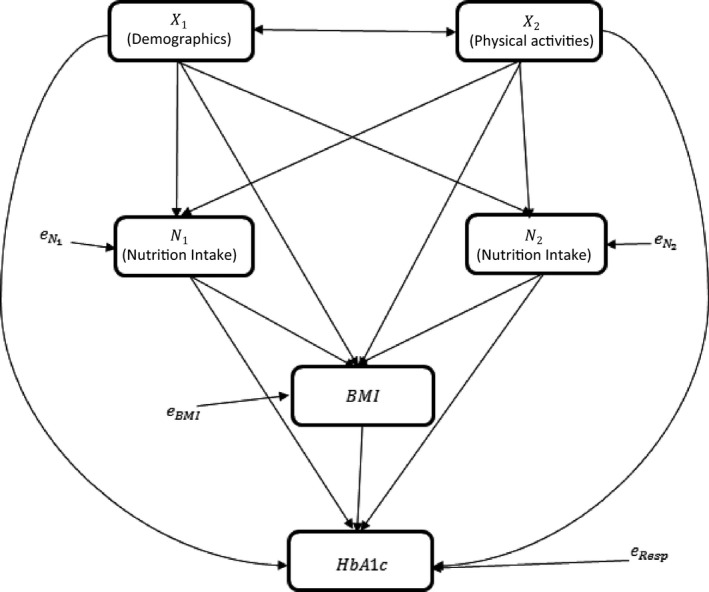
Hypothetical causal model:^14^ *X*_1_ and *X*_2_ indicate demographic characteristics (including age, gender and ethnicities) and physical activity, respectively, and *N*_1_ and *N*_2_ are nutrition intake variables.

Path analysis^14^, ^15^, ^16^ is used to estimate the causal relationships between demographic variables, physical activity and dietary information to the outcome variable, HbA1c. HbA1c indicates T2DM control, with well‐controlled T2DM (ie < 6.5%) or poorly controlled diabetes (8.0% or over). We hypothesize that demographic characteristics (*X_1_*) and physical activity (*X_2_*) affect nutrition intake (*N_1_* and *N_2_*), which in turn affects BMI and HbA1c. Physical activity and nutrition intake also directly affect HbA1c Figure 1. The demographic and the physical activity variables (*X_s_*) are exogenous variables (ie variances not explained by variables in the hypothetical causal model). The nutritional variables (*N_s_*), BMI and HbA1c are endogenous variables (ie variances are partially explained by the other variables within the causal model). In the path diagram, a double‐headed arrow indicates a correlation between two variables that are not causally related. Single‐headed arrows indicate causal paths.^14^, ^15^, ^16^

Path analysis has not been used previously to analyse the relationship of physical activity and nutrition to HbA1c. Path analyses use causal structural equation models^14^, ^15^, ^16^ to fit a multivariate nonexperimental dataset to a complex causal model. The path coefficient (*ρ*) is determined by fitting the causal structural equations and the significance level is adjusted using Bonferroni corrections.^16^ Path analysis is a powerful technique to analyse and compare different complex hypothetical causal models, and to find the model with the most consistent fit to the data.^15^, ^16^ Thus, the causal importance of the variables in the pathways to the outcomes can be quantitatively estimated. Due to the large number of nutrition components in the NHANES data, we carried out preliminary regression analyses by regressing HbA1c on all demographic variables and nutrition components to obtain a small set of nutrition components which had significant direct effect on HbA1c. These variables were remained for the path analyses.

In the causal model Figure 1, four different paths from demographic variables or physical activity may hypothetically affect the outcome, HbA1c: (a) direct effect: X → HbA1c, (b) indirect effect through nutrition intake: X → N → HbA1c; (c) indirect effect through BMI: X → BMI →HbA1c; and (d) indirect effect through nutrition intake and BMI: X → N → BMI → HbA1c. Structural equations for endogenous variables were obtained using multivariate linear regression model using standardized continuous variables (mean = 0, SD = 1.0). Path coefficient estimates (*ρ*) are the resulting beta regression coefficients by regressing each endogenous variable on its causal variables.^15^, ^16^ Path coefficient significance is adjusted by Bonferroni correction for multiple comparisons. The direct effect from X → HbA1c is the path coefficient for X in the structural equation model for HbA1c. Indirect effects are estimated as the product of the path coefficients (*ρ*_1_ * *ρ*_2_) on the specified pathway. The total causal effects of demographic variables or physical activity on the outcome are the sum of the direct and indirect effects from all the paths that lead to the outcome, HbA1c.^16^

In the path analysis, all path coefficients (*ρ*) were estimated, and the significance of each path coefficient p value was adjusted using the Bonferroni method. Path coefficients (*ρ*) fall into the interval (−1, 1). The larger the path coefficient's absolute value is, the stronger causation. A positive path coefficient indicates that a causal variable increases the outcome value, and a negative path coefficient indicates a causal variable decreases the outcome value. Only significant paths (Bonferroni adjusted *P*‐value < 0.05) were retained. An indirect path (ie the path that goes through one or more mediators) was considered significant if the absolute value of the product of the single path coefficients is greater than 0.01 using the path product property.^15^, ^16^ Path analysis was performed for the entire population first, and then in each homogeneous group (gender‐specific and gender‐ethnicity‐specific). Path analyses were performed using R statistical software with package ‘lavaan’.^21^

## 3 RESULTS

The descriptive statistics of variables included in the path analysis are reported in Table 1. The study cohort included 2831 subjects with average age of 48.1 ± 16.2 years. 52.7% of the participants were female, and 47.3% were male. The study population included three major ethnicities in the United States: 1572 (55.5%) non‐Hispanic white, 814 (28.7%) non‐Hispanic Black, and 445 (15.7%) Mexican Americans (Hispanic). The multivariate linear model regression of HbA1c on all demographic variables, physical activity, 65 nutritional variables and BMI was done, and significant nutritional variables were identified. Seven significant nutritional variables were significant (*P* < .05), including protein, carbohydrate, total sugars, total fat, Mg, moisture and butanoic fatty acid. The full model included 73 covariates with an unadjusted *R* ^2^ at 0.1736 and the adjusted *R* ^2^ at 0.1517 (*F*‐test *P*‐value < 0.0001). The reduced model included 15 covariates with an unadjusted *R* ^2^ at 0.1526 and an adjusted *R* ^2^ at 0.1480 (*F*‐test *P*‐value < 0.0001).

**Figure 2:**
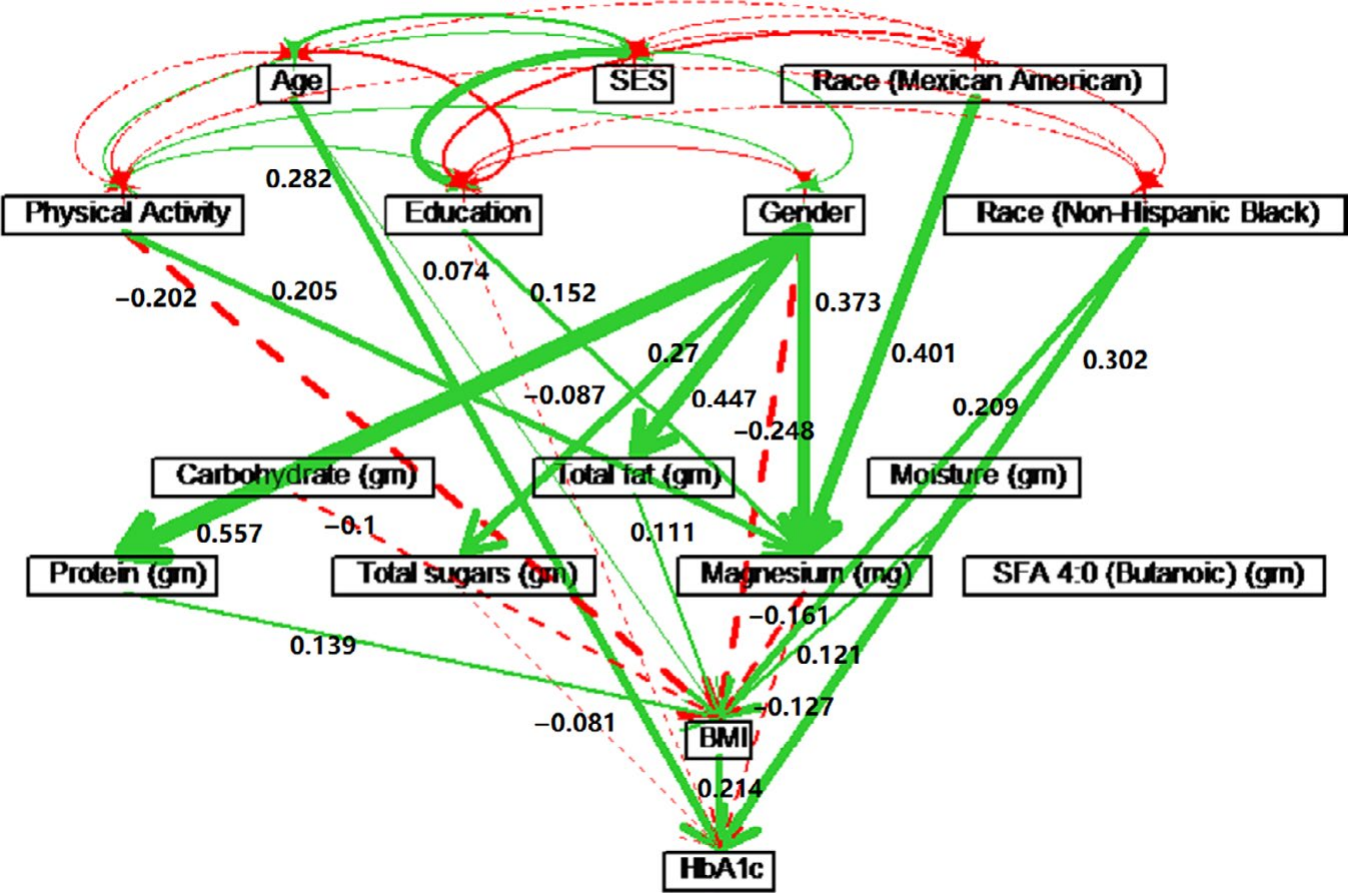
Path diagram for the entire sample. Paths of solid lines (green) indicate positive path coefficients, while paths of dashed lines (red) indicate negative path coefficients. The widths of the paths are related to the absolute values of path coefficients, and the wider of the line is the stronger causation. Only significant paths are shown in the diagram.

The path diagram Figure 2 showed the significant paths, and Table 2 showed the estimated significant path coefficients. Physical activity did not have a significant direct causal effect on HbA1c but had a significant effect on HbA1c indirectly through BMI and Mg intake. Through BMI, physical activity had an effect of *ρ* = −0.043 (=−0.202 × 0.214) on decreasing HbA1c, and through Mg intake, the effect was *ρ* = −0.026 (=0.205 × (−0.125)). Hence, physical activity had a total effect of *ρ* = −0.069 on decreasing HbA1c.

**Table 2:** Significant paths and summarized causal effects based on the path analysis for the entire population. A bold value indicates the total effect (both direct and indirect) on HbA1c.

Paths	Intermediate Path Coefficients			Total Direct/Indirect Effects
	*ρ*_1_	*ρ*_2_	*ρ*_3_	
Physical activity				
Physical Activity ‐> BMI				−0.202
Physical Activity ‐> Magnesium				0.205
Physical Activity ‐> Moisture				0.184
Physical Activity ‐> BMI ‐> HbA1c		−0.202	0.214	−0.043
Physical Activity ‐> Magnesium ‐> HbA1c		0.205	−0.127	−0.026
Total effects on HbA1c				−**0.069**
Age				
Age ‐> HbA1c				0.282
Age ‐> BMI				0.074
Age ‐> Protein				−0.086
Age ‐> Carbohydrate				−0.124
Age ‐> Total_Sugars				−0.110
Age ‐> Total_Fat				−0.084
Age ‐> SFA_40_Butanoic				−0.085
Age ‐> BMI ‐> HbA1c		0.074	0.214	0.016
Total effects on HbA1c				**0.298**
Education				
Education ‐> HbA1c				−0.087
Education ‐> Protein				0.074
Education ‐> Magnesium				0.152
Education ‐> Moisture				0.078
Education ‐> Magnesium ‐> HbA1c		0.152	−0.127	−0.019
Total effects on HbA1c				−**0.106**
Gender (1 for male, and 0 for female)				
Gender ‐> BMI				−0.248
Gender ‐> Protein				0.557
Gender ‐> Carbohydrate				0.442
Gender ‐> Total_Sugars				0.270
Gender ‐> Total_Fat				0.447
Gender ‐> Magnesium				0.373
Gender ‐> Moisture				0.261
Gender ‐> SFA_40_Butanoic				0.205
Gender ‐> BMI ‐> HbA1c		−0.248	0.214	−0.053
Gender ‐> Total_Sugars ‐> HbA1c		0.270	−0.081	−0.022
Gender ‐> Magnesium ‐> HbA1c		0.373	−0.127	−0.047
Gender ‐> Protein ‐> BMI ‐> HbA1c	0.557	0.139	0.214	0.017
Gender ‐> Total_Fat ‐> BMI ‐> HbA1c	0.447	0.111	0.214	0.011
Gender ‐> Magnesium ‐> BMI ‐> HbA1c	0.373	−0.161	0.214	−0.013
Total effects on HbA1c				−**0.107**
Mexican Americans				
MexA ‐> Protein				0.295
MexA ‐> Magnesium				0.401
MexA ‐> Magnesium ‐> HbA1c		0.401	−0.127	−0.051
MexA ‐> Magnesium ‐> BMI ‐> HbA1c	0.401	−0.161	0.214	−0.014
Total effects on HbA1c				−**0.065**
Non‐Hispanic Black				
NHB ‐> HbA1c				0.302
NHB ‐> BMI				0.209
NHB ‐> Moisture				−0.359
NHB ‐> SFA_40_Butanoic				−0.266
NHB ‐> BMI ‐> HbA1c		0.209	0.214	0.045
Total effects on HbA1c				**0.347**
Total Sugars				
Total Sugars ‐> HbA1c				−**0.081**
Protein				
Protein ‐> BMI				0.139
Protein ‐> BMI ‐> HbA1c		0.139	0.214	**0.030**
Carbohydrate				
Carbohydrate ‐> BMI				−0.100
Carbohydrate ‐> BMI ‐> HbA1c		−0.100	0.214	−**0.021**
Total Fat				
Total Fat ‐> BMI				0.111
Total Fat ‐> BMI ‐> HbA1c		0.111	0.214	**0.024**
Magnesium				
Magnesium ‐> HbA1c				−0.127
Magnesium ‐> BMI				−0.161
Magnesium ‐> BMI ‐> HbA1c		−0.161	0.214	−0.034
Total effects on HbA1c				−**0.161**
Moisture				
Moisture ‐> BMI				0.121
Moisture ‐> BMI ‐> HbA1c		0.121	0.214	0.026
BMI				
BMI ‐> HbA1c				**0.214**

Note

Nutritional variables in the path analysis include protein, total sugars, total fat, magnesium, moisture and butanoic fatty acid. Only significant direct effects and indirect effects are reported, and the intermediate path coefficients for indirect effects are reported under the column headings *ρ*_1_, *ρ*_2_ and *ρ*_3_.

Age had increased HbA1c with a total causal effect of *ρ* = 0.298, which included a direct effect of *ρ* = 0.282 and an indirect effect of *ρ* = 0.016 through BMI. Similarly, a higher education level caused a decrease in HbA1c level with a direct effect of *ρ* = −0.087 and an indirect effect of *ρ *= −0.019 through Mg intake, resulting in a total causal effect (*ρ* = −0.106) on HbA1c. Socioeconomic status effect on HbA1c was not significant.

Among the seven nutritional variables included in the path analysis, butanoic fatty acid did not have a significant causal relationship with HbA1c. Total sugar intake had a direct effect of *ρ* = −0.081 on decreasing HbA1c. Protein, carbohydrate, total fat and moisture had indirect effects on HbA1c through BMI, which were *ρ* = 0.030, −0.021, 0.024 and 0.026, respectively. Higher Mg intake had lowered HbA1c significantly (*ρ* = −0.161), which included a direct effect of *ρ* = −0.127 and an indirect effect of *ρ* = −0.034 through the BMI path.

**Figure 3:**
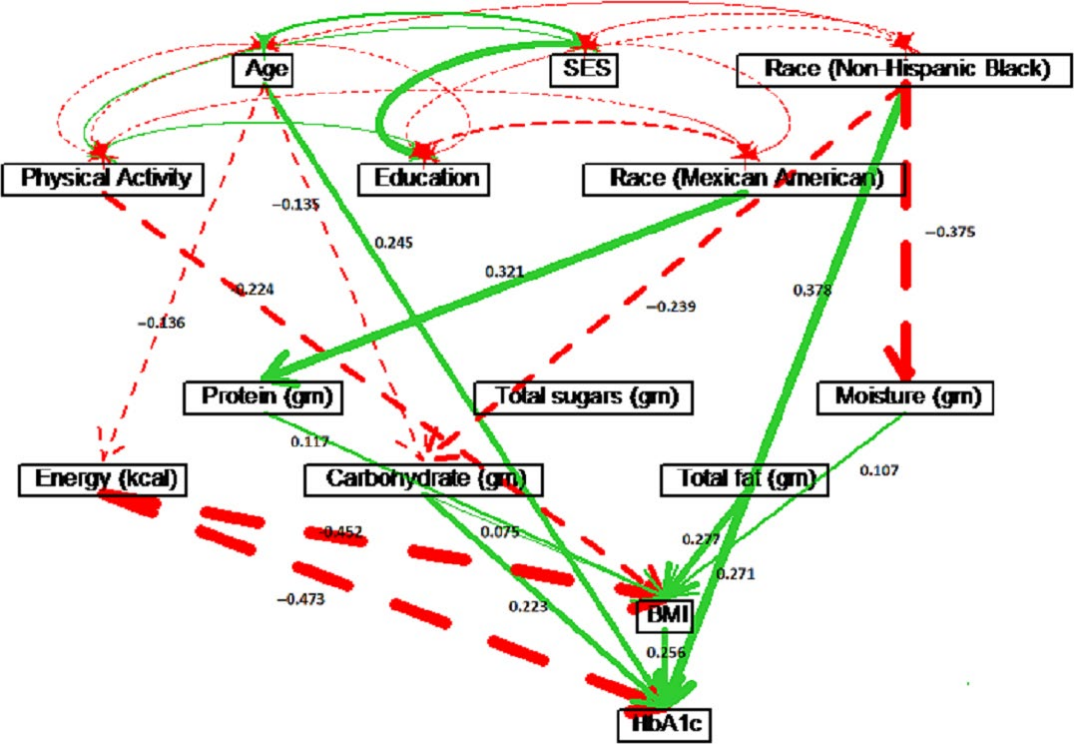
Path diagram for males only. Solid (green) paths indicate positive path coefficients, while dashed (red) paths indicate negative path coefficients. The widths of the paths are related to the absolute values of path coefficients, where wider path indicates higher absolute path coefficient. Only significant paths are shown in the diagram

**Figure 4:**
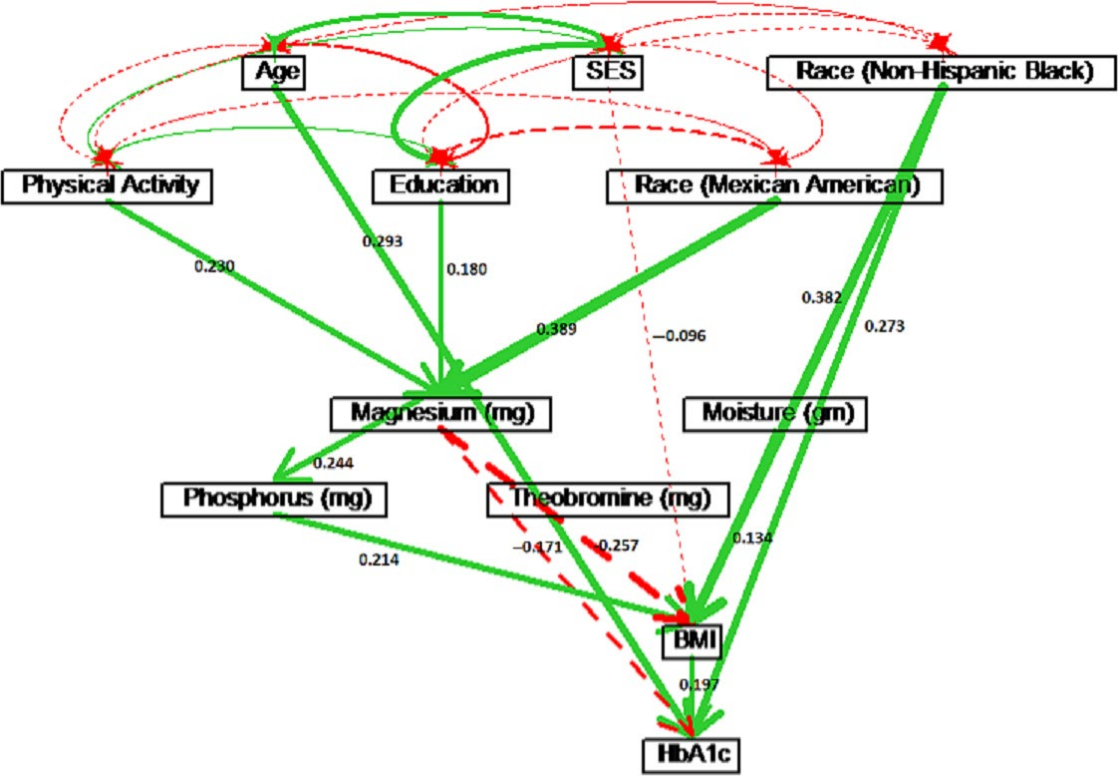
Path diagram for females only. Solid (green) paths indicate positive path coefficients, while dashed (red) paths indicate negative path coefficients. The width of the paths is related to the absolute values of path coefficients, where wider path indicates higher absolute path coefficient. Only significant paths are shown in the diagram.

Gender played an important role in the causal model. Although gender did not affect HbA1c directly, gender indirectly affected HbA1c through one or two intermediate variables, particularly through BMI and Mg intake (see Table 2). To further investigate the difference in the causal structure between male and female, separate path analyses were performed for males and females (see Figure 3 and Table S1 for males, and Figure 4 and Table S2 for females). Physical activity had similar causal effects on HbA1c for both males (*ρ* = −0.057) and females (*ρ* = −0.051). However, physical activity affected HbA1c through BMI for males, while physical activity affected HbA1c through Mg intake for females. For females, age had a direct effect of *ρ* = 0.293 on HbA1c. For males, age had a direct effect of *ρ* = 0.245 on HbA1c, and age also affected HbA1c via energy and carbohydrate intake with a total effect of *ρ* = 0.295. Education and SES did not have significant causal effects on HbA1c for males. However, for females, higher education level indicated higher Mg intake, which further decreased HbA1c level (*ρ* = −0.031). For females, higher SES level had lowered HbA1c through BMI (*ρ* = −0.019), indicating that lower SES caused higher BMI, which led to a higher HbA1c value.

**Figure 5:**
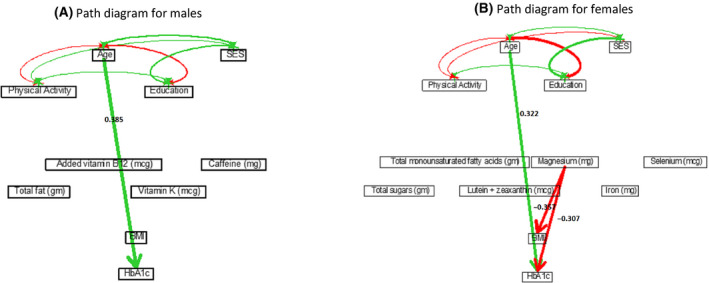
Path diagrams for Mexican American (males on the left, females on the right). Solid (green) paths indicate positive path coefficients, while dashed (red) paths indicate negative path coefficients. The widths of the paths are related to the absolute values of path coefficients, where wider path indicates higher absolute path coefficient. Only significant paths are shown in the diagram.

**Figure 6:**
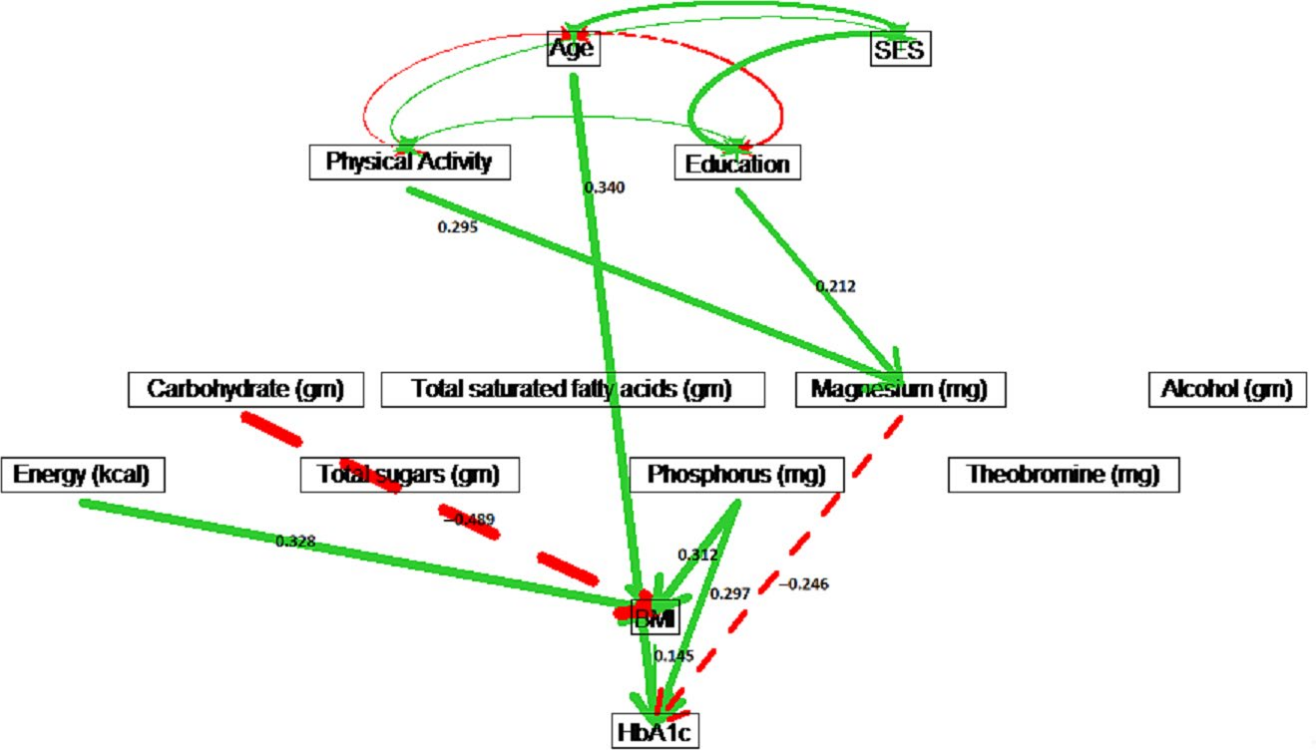
Path diagram for non‐Hispanic black females. Solid (green) paths indicate positive path coefficients, while dashed (red) paths indicate negative path coefficients. The widths of the paths are related to the absolute values of path coefficients, where wider path indicates higher absolute path coefficient. Only significant paths are shown in the diagram

**Figure 7:**
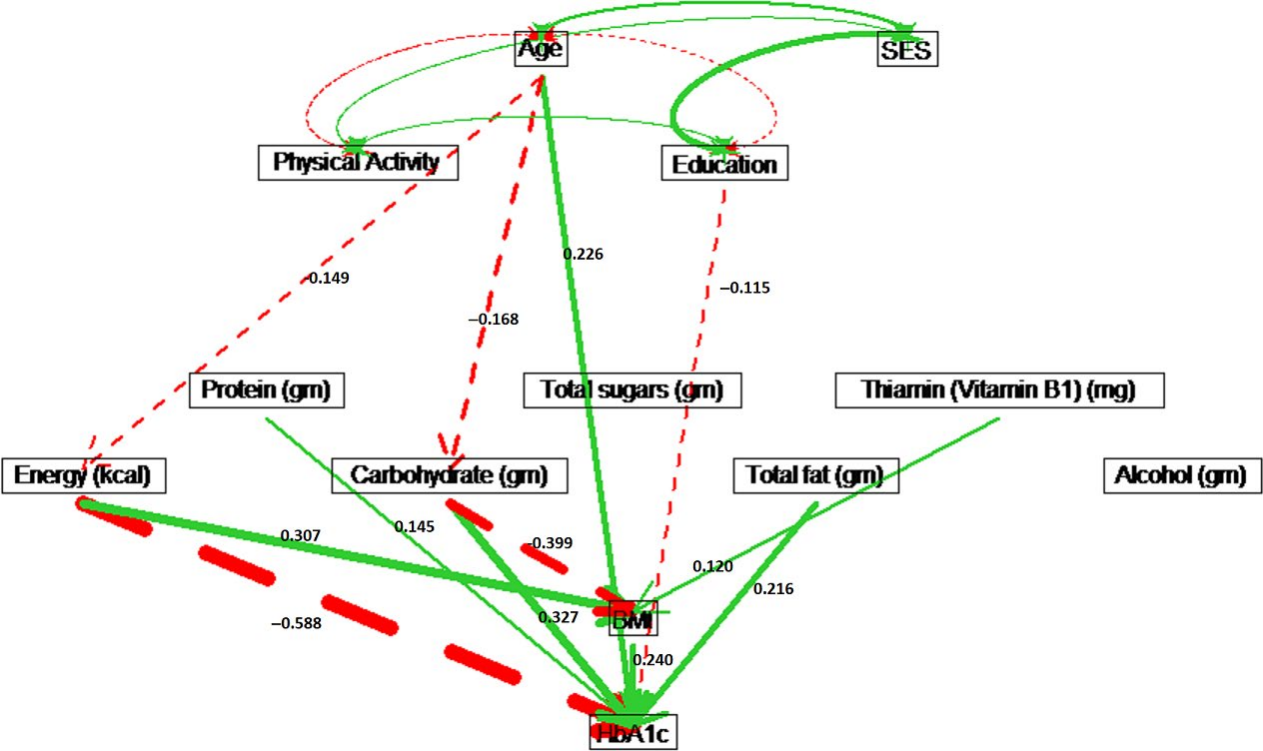
Path diagram for non‐Hispanic white males. Solid (green) paths indicate positive path coefficients, while dashed (red) paths indicate negative path coefficients. The widths of the paths are related to the absolute values of path coefficients, where wider path indicates higher absolute path coefficient. Only significant paths are shown in the diagram.

**Figure 8:**
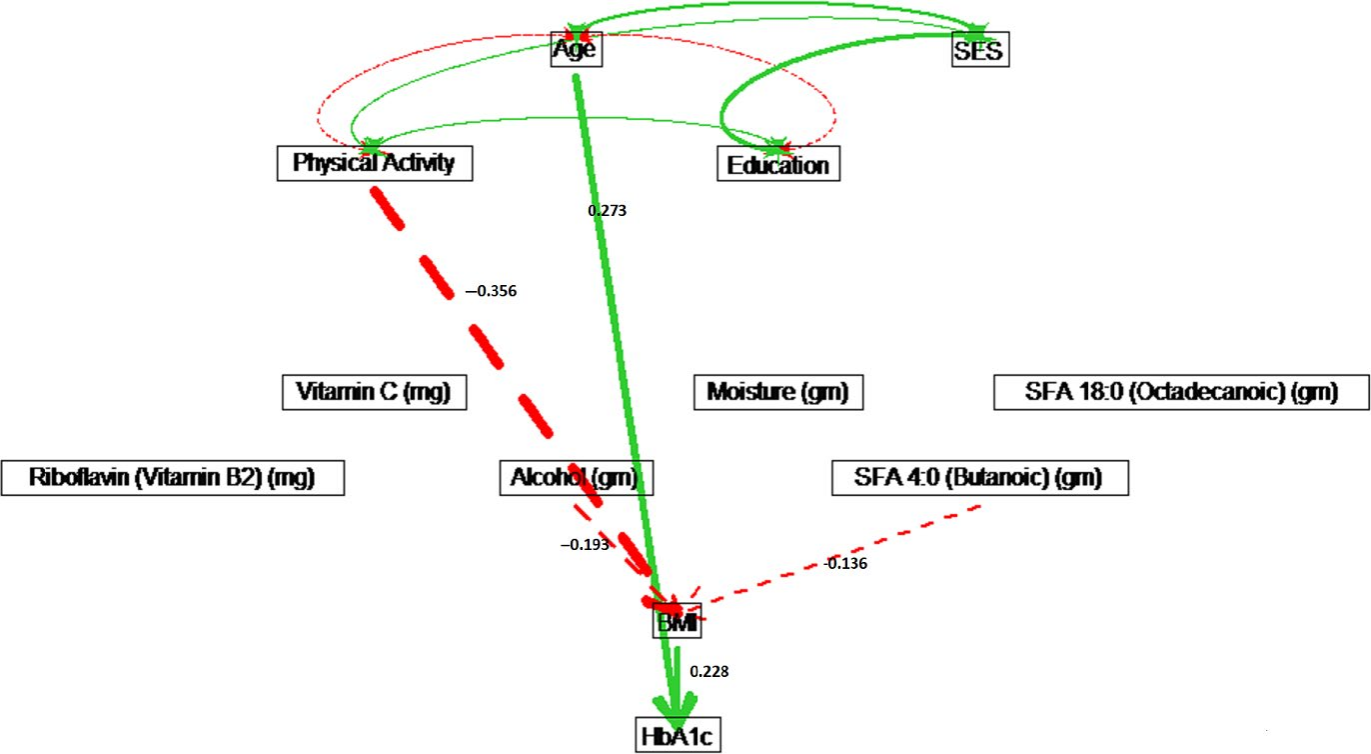
Path diagram for non‐Hispanic white females. Solid (green) paths indicate positive path coefficients, while dashed (red) paths indicate negative path coefficients. The widths of the paths are related to the absolute values of path coefficients, where wider path indicates higher absolute path coefficient. Only significant paths are shown in the diagram.

Based on our study, Mexican American ethnicity had a causal effect on decreasing HbA1c (*ρ* = −0.065, Table 2) through Mg intake. Non‐Hispanic black ethnicity had a strong causal effect on increasing HbA1c (*ρ* = 0.347), which included a direct effect of *ρ* = 0.302 and an indirect effect of *ρ* = 0.045 through BMI. Analysis of the causal relationships specific to each homogeneous ethnic group was done in separate path analyses for each gender‐ethnicity‐specific group (see Tables S3‐S5 in the supplement, and Figures 5, 6, 7, 8). Based on the path analysis on different gender‐ethnicity‐specific subpopulations, significant nutritional variables in the path analysis for Mexican American males included total fat, vitamin B12 supplements, vitamin K and caffeine (see Figure 5). Significant nutritional variables included for Mexican American females were total sugar intake, total monounsaturated fatty acids, lutein and zeaxanthin, Mg, iron and selenium. Age had a strong direct effect of *ρ* = 0.385 on increasing HbA1c for Mexican American males, not substantively differing from *ρ* = 0.322 for Mexican American females. Education and SES did not have any significant causal effects on HbA1c for Mexican Americans (see Figure 5 and Table S3 in the supplement).

Estimated path coefficients for the non‐Hispanic black males (see Table S4 in the supplement) showed that nutritional variables were not significant for HbA1c. For non‐Hispanic black females, physical activity had a significant protective effect on HbA1c (*ρ* = −0.073) through Mg intake. Similarly, education also had reduced HbA1c (*ρ* = −0.052) through Mg intake. Age had a strong direct effect on increasing HbA1c (*ρ* = 0.340) for non‐Hispanic black females (see Figure 6).

For non‐Hispanic white males, age had a causal effect of *ρ* = 0.264 on increasing HbA1c, which included a direct effect of *ρ* = 0.226, and indirect effects through energy intake, carbohydrate intake and BMI (see Figure 7). For non‐Hispanic white females, only age had a significant direct effect of *ρ* = 0.273 on HbA1c (see Figure 8). For white males, education had a direct effect of *ρ* = −0.115 on HbA1c, while education was not significant for white females. However, physical activity lowered BMI and resulted in an indirect effect of *ρ* = −0.081 on HbA1c for white females (see Figure 8 and Table S5 in the Supplement). Physical activity had no significant effect on HbA1c for white males (see Figure 7 and Table S5).

In summary, paths to HbA1c varied by ethnicity and gender. A consistent finding in the combined groups analysis, and across ethnic and gender groups, higher Mg levels caused lower HbA1c levels, directly and indirectly. Notably, physical activity increased Mg intake (*ρ* = 0.205, Table  2), and higher Mg dietary intake decreased HbA1c though BMI (*ρ* = −0.161*0.214= −0.034). The direct path revealed that as Mg intake increased, BMI tended to decrease (*ρ* = −0.161, Table 2). Accordingly, lower BMI caused lower HbA1c (*ρ* = 0.214). The effect of BMI on HbA1 was stronger among males (*ρ* = 0.256, see Tables S1) than among female (*ρ* = 0.197, see Tables S2).

## 4 DISCUSSION AND CONCLUSION

Age was consistently significantly increased HbA1c in all analyses. The relationship between BMI and T2DM has been well investigated.^11^, ^12^ BMI is a modifiable risk factor for T2DM. A clear causal association between BMI and T2DM in a large, representative sample of the US population using NHANES 1999‐2006 data is documented.^11^ The lowest prevalence of diabetes was found in the normal weight group (BMI < 25.0), and the prevalence of diabetes increased as BMI increased.^11^ Our study showed a strong direct effect of BMI on increasing HbA1c across all gender‐ethnicity subgroups. Some dietary intake factors (eg protein and carbohydrate) affected HbA1c only through BMI. In order to clarify causal paths through BMI, additional path analyses that used two dummy variables (overweight if 25 ≤ BMI<30; obesity if BMI ≥ 30) indicated that all the causal effects on HbA1c from independent variables through BMI were through obesity (see Figure S2 in the Supplement). These findings agreed with previously published results^11^ that the prevalence of diabetes increased as the severity of obesity increased. Our study found higher BMI was associated with increased HbA1c. The indirect effect from physical activity through obesity but not overweight to HbA1c is also supported by the finding that physical activity lowered HbA1c in subjects with high BMI. Literature^22^ suggests that adjusting for total energy intake is important. The use of BMI as an intermediary variable between nutrients and HbA1c in the present model apparently was a proxy for total energy intake. In this analytical approach, total energy expenditure or its derivatives were not significant (*P* > .05) and dropped from the subsequent path analysis.

In the present study, the effect of physical activity on HbA1c varied across different homogeneous groups (gender, ethnicity). The total effect of physical activity on HbA1c was through several indirect paths through dietary intake. Males had higher path coefficients for intake of all nutritional components than females, especially for protein and total fat. However, females had a higher BMI (*ρ* = −0.248, Table 2) causing a higher HbA1c, compared with males. Differences in body composition between females and males may explain this difference (ie a higher per cent body fat in females than males). Non‐Hispanic blacks had a higher HbA1c compared to non‐Hispanic whites (*ρ* = 0.347, Table 2). However, in this study, Mexican Americans had a lower HbA1c than non‐Hispanic white (*ρ* = −0.065) mediated through Mg intake.

Our study revealed the importance of Mg intake and physical activity in decreasing BMI and HbA1c. Physical activity increased Mg intake (*ρ* = 0.205, Table 2). Higher Mg intake lowered HbA1c significantly (*ρ* = −0.161, Table 2), particularly in females (*ρ* = −0.478, Table S2 in the Supplement). Increased Mg intake caused lower BMI (*ρ* = −0.161, Table 2) and lower BMI caused lower HbA1c (*ρ* = 0.214, Table 2). The path coefficient from Mg intake to HbA1c was large (*ρ* = −0.127) and highly significant (*P* < .0001), indicating that the relationship of Mg intake and HbA1c is a robust and true observation, not by chance. The mean and standard deviation for Mg levels across different variables in Table 3 provided additional evidence for the strong association between Mg intake and BMI, ethnicity, gender and physical activity in control of HbA1c level. Therefore, the present study indicated the relationships between physical activity and Mg intake were statistically significant and biologically plausible predictors of HbA1c across all ethnic and gender subgroups. The causal association between physical activity, Mg intake and HbA1c control was supported by prior research.^12^, ^23^, ^24^

**Table 3:** Mean and standard deviation (SD) of magnesium intake (mg/day) stratified by HbA1c levels and other important variables (ie BMI, Ethnicity, Gender and Physical activity)

		Total	HbA1c < 6.5%	6.5%≤HbA1c < 8.0%	HbA1c ≥ 8.0%	*P*‐value^b^
Sample size (N)		2831	2517	194	120	
		Mean (SD)	Mean (SD)	Mean (SD)	Mean (SD)	
Overall		291.8 (153.9)	295.4 (155.8)	270.5 (144.7)	250.5 (115.5)	.001
BMI^a^	Normal (BMI < 25)	305.2 (173.9)	307.5 (175.6)	262.8 (89.9)	204.6 (131.6)	.126
	Overweight (25 ≤ BMI<30)	296.1 (153.1)	296.8 (152.6)	291.1 (177.1)	276 (124.8)	.810
	Obesity (BMI ≥ 30)	280.7 (140.5)	285.7 (142.6)	265.3 (139.6)	249.2 (111.4)	.025
Ethnicity^a^	Mexican American	331.4 (172.2)	337.5 (176.4)	301.7 (145.9)	262.8 (99.0)	.092
	Non‐Hispanic Black	265.6 (144.6)	267.5 (143.9)	260.1 (159.5)	247.6 (129.4)	.631
	Non‐Hispanic White	294.2 (150.5)	297.4 (152.6)	268.4 (128.5)	248.2 (110.8)	.017
Gender^a^	Male	323.1 (173.2)	326.9 (175.4)	306.8 (166.8)	275.0 (128.5)	.045
	Female	263.8 (127.9)	267.6 (130.0)	235.7 (109.6)	224.4 (93.9)	.003
Physical Activity^a^	Yes	305.1 (159.9)	308.0 (160.6)	285.3 (166.1)	262.1 (121.0)	.028
	No	260.9 (134.0)	264.0 (138.3)	250.7 (107.5)	235.8 (107.4)	.259

^a^ indicates that the variable had significant effect on magnesium intake;

^b^ indicates the p‐value for testing whether the magnesium levels are significantly different among the three groups (ie, HbA1c < 6.5%, 6.5% ≤ HbA1c < 8.0% and HbA1c ≥ 8.0%).

Dietary intake of Mg was higher among those who were more physically active, and both factors were associated with lower HbA1c.^25^, ^26^ Meta‐analyses of more than 20 investigations showed that increased Mg intake was associated with higher physical activity and lower HbA1c.^27^, ^28^, ^29^ Higher physical activity and Mg intake were associated with better glycemic control and lower BMI.^30^, ^31^ These published findings aligned with the causal models developed in the present investigation in which physical activity was associated with higher Mg intake, lower BMI and decreased HbA1c. Mg intake was also associated with lower levels of inflammation markers in diabetics^32^ ^,^ and with reduced comorbidities in diabetics such as myocardial infarction.^33^ Ultimately, one benefit on increased Mg intake was decreased insulin resistance/ increased sensitivity.^34^

**Figure 9:**
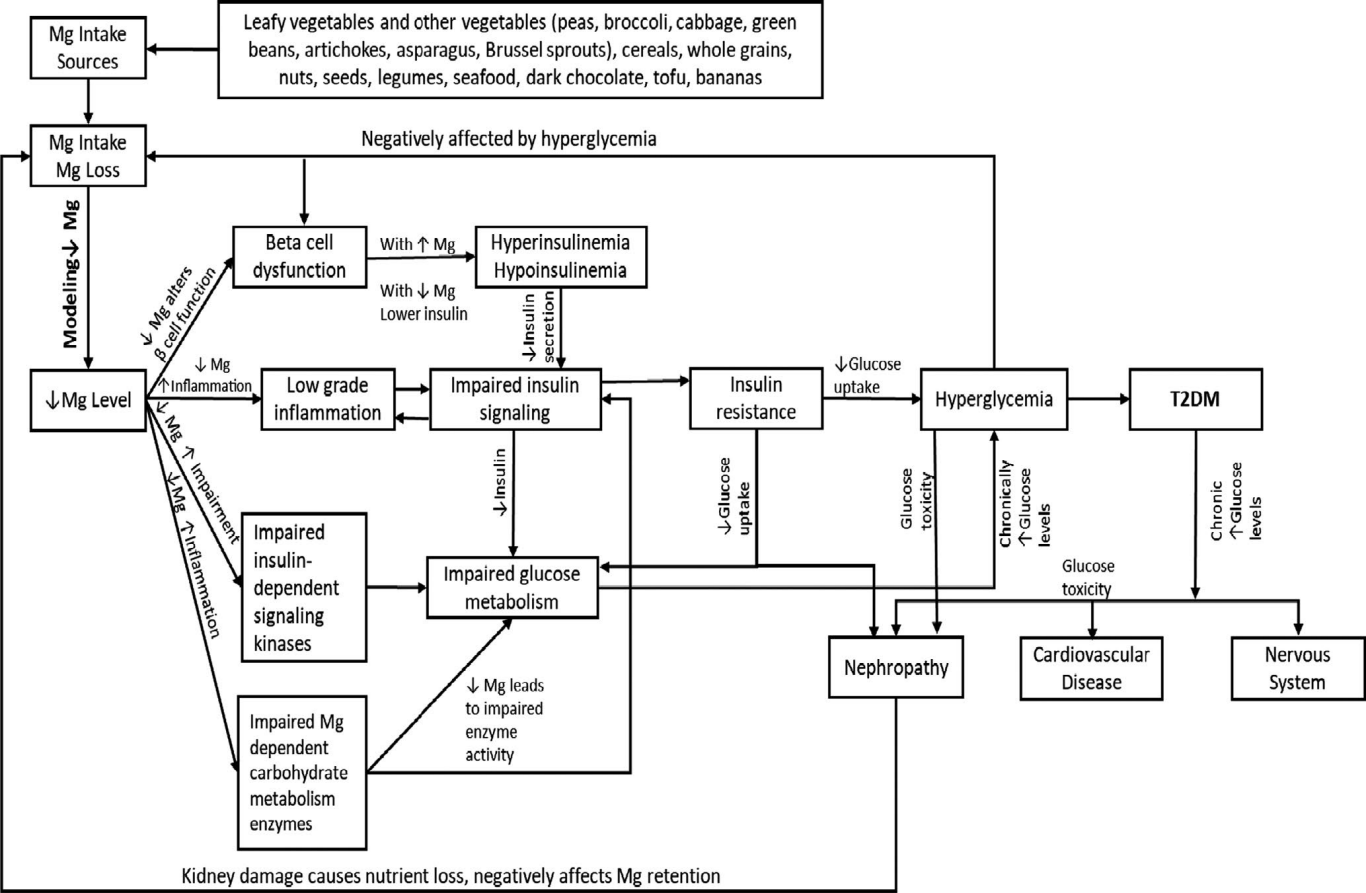
Relationship between magnesium intake and T2DM (modified from^35^ ^,^ ^40^)

The mechanisms that underly the apparent benefit of Mg in type 2 diabetes involve several pathways (see Figure 9). Lower Mg intake is associated with poorer beta‐cell insulin secretion, which may be partly compensated through Mg supplementation.^35^, ^36^ Hypomagnesemia causes impaired carbohydrate and other nutrient metabolism.^37^, ^38^ Inflammatory markers synergistically impaired insulin signalling, contributing to insulin resistance (see double‐headed arrows in Figure 9).^39^, ^40^ Mg plays an important role in T2DM glucose control. The patients with T2DM should take the foods with good sources of Mg, which include leafy vegetables and other vegetables (peas, broccoli, cabbage, green beans, artichokes, asparagus, Brussel sprouts), cereals, whole grains, nuts, seeds, legumes, seafood, dark chocolate, tofu and bananas (see Figure 9).

In summary, multivariate causal analyses of the causal relationship of the two important components (physical activity and nutrition) on HbA1c were carried out in a T2DM population, as well as in different homogeneous subgroups. Variability in causal structures was observed across different subgroups, while the causal importance of some components such as the intake of Mg remained important across all homogeneous gender‐ethnicity subgroups. Mg intake was a significantly protective factor, decreasing HbA1c. Our work provides insight into the causal association of physical activity and Mg with lower HbA1c. Future studies are needed to better understand the role of Mg intake and supplementation as preventative for T2DM, and better elucidate the Mg's metabolic role in normal and T2DM metabolism.

This study has several limitations. The study cohort was limited to T2DM individuals, which was confirmed by oral hypoglycemic agent use. Those subjects with missing values on any one of the analytical variables were excluded, and we assumed that the missing data were missing at random. The NHANES 2011‐2014 data provided physical activity (PA) intensity information, but not the duration. The NHANES physical activity questionnaire data defined PA as physically active or not. A subject was defined as physically active if the subject reported either vigorous or moderate weekly work/recreational activities. The NHANES dietary data are based on self‐report of food and liquid intake. From these data, nutrient intake is estimated after adjusting for day‐to‐day variation. A further limitation is that the quantity of food intake must also be estimated, further confounding the data. Therefore, the actual measurement of dietary intake did not occur, and it was self‐reported intake and quantity using cups, spoon and rulers provided by NHANES.

The limitations of path analysis include (a) relations among variables in the model are assumed to be linear, additive and causal; b) there is one‐way causal flow, that is, reciprocal causation between variables is ruled out; and c) the variables are measured on an numerical scale and are measured without error. Regardless of these limitations, the findings here for the effect of Mg intake and physical activity on control of glucose in T2DM are robust.

## 5 ETHICS STATEMENT

NHANES data are collected by the US Public Health Service for the purpose of surveillance research. The NHANES datasets are completely anonymized and contain no protected health information (PHI). The IRB classification of this data is Exempt and is in accordance with the Declaration of Helsinki.

## CONFLICT OF INTEREST

The authors declare that the research was conducted in the absence of any commercial or financial relationships that could be construed as a potential conflict of interest.

## AUTHOR CONTRIBUTIONS

You Wu, Susmita Datta, Bert Little and Maiying Kong contributed to the analysis and interpretation of results. You Wu conducted all data manipulations as the study data was a proprietary secured database.

## Supporting information

Supplementary Material

## Data Availability

NHANES data are collected by the US Public Health Service for the purpose of surveillance research. The NHANES datasets are completely anonymized and contain no protected health information (PHI). The IRB classification of this data is Exempt and is in accordance with the Declaration of Helsinki.
