# Supplementary material for: Magnesium dietary intake and physical activity in Type 2 diabetes by gender in White, African‐American and Mexican American: NHANES 2011‐2014
Source: Endocrinol Diabetes Metab. 2020 Nov 20;4(1):e00203. doi: 10.1002/edm2.203 (PMC7831210; doi:10.1002/edm2.203)
Supplement: Supplementary file 1 — Supplementary Material [file EDM2-4-e00203-s001.docx]

**Supplementary Materials**

**Table S1** Results for males only: significant paths and summarized causal effects for each variable in the path analysis. Dietary variables include energy, protein, carbohydrate, total sugars, total fat and moisture. For indirect effects, intermediate path coefficients are reported under the columns of ρ1, ρ2, and ρ3.

| **Paths** | **Intermediate Path Coefficients** | | | **Total Direct/Indirect Effects** |
| --- | --- | --- | --- | --- |
|  | **ρ1** | **ρ2** | **ρ3** |  |
| **Physical Activity** |  |  |  |  |
| Physical Activity -> BMI |  |  |  | -0.224 |
| Physical Activity -> Moisture |  |  |  | 0.239 |
| Physical Activity -> BMI -> HbA1c |  | -0.224 | 0.256 | -0.057 |
| Total effects on HbA1c |  |  |  | **-0.057** |
| **Age** |  |  |  |  |
| Age -> HbA1c |  |  |  | 0.245 |
| Age -> Energy |  |  |  | -0.136 |
| Age -> Carbohydrate |  |  |  | -0.135 |
| Age -> Total Sugars |  |  |  | -0.121 |
| Age -> Energy -> HbA1c |  | -0.136 | -0.473 | 0.064 |
| Age -> Carbohydrate -> HbA1c |  | -0.135 | 0.223 | -0.030 |
| Age -> Energy -> BMI -> HbA1c | -0.136 | -0.452 | 0.256 | 0.016 |
| Total effects on HbA1c |  |  |  | **0.295** |
| **Mexican American** |  |  |  |  |
| MexA -> Protein |  |  |  | 0.321 |
| MexA -> Protein -> BMI -> HbA1c | 0.321 | 0.117 | 0.256 | **0.010** |
| **Non-Hispanic Black** |  |  |  |  |
| NHB -> HbA1c |  |  |  | 0.378 |
| NHB -> Carbohydrate |  |  |  | -0.239 |
| NHB -> Total_Sugars |  |  |  | -0.233 |
| NHB -> Moisture |  |  |  | -0.375 |
| NHB -> Carbohydrate -> HbA1c |  | -0.239 | 0.223 | -0.053 |
| NHB -> Moisture -> BMI -> HbA1c | -0.375 | 0.107 | 0.256 | -0.010 |
| Total effects on HbA1c |  |  |  | **0.315** |
| **Energy** |  |  |  |  |
| Energy -> HbA1c |  |  |  | -0.473 |
| Energy -> BMI |  |  |  | -0.452 |
| Energy -> BMI -> HbA1c |  | -0.452 | 0.256 | -0.116 |
| Total effects on HbA1c |  |  |  | **-0.589** |
| **Protein** |  |  |  |  |
| Protein -> BMI |  |  |  | 0.117 |
| Protein -> BMI -> HbA1c |  | 0.117 | 0.256 | **0.030** |
| **Carbohydrate** |  |  |  |  |
| Carbohydrate -> HbA1c |  |  |  | 0.223 |
| Carbohydrate -> BMI |  |  |  | 0.075 |
| Carbohydrate -> BMI -> HbA1c |  | 0.075 | 0.256 | 0.019 |
| Total effects on HbA1c |  |  |  | **0.242** |
| **Total Fat** |  |  |  |  |
| Total Fat -> HbA1c |  |  |  | 0.271 |
| Total Fat -> BMI |  |  |  | 0.277 |
| Total Fat -> BMI -> HbA1c |  | 0.277 | 0.256 | 0.071 |
| Total effects on HbA1c |  |  |  | **0.342** |
| **Moisture** |  |  |  |  |
| Moisture -> BMI |  |  |  | 0.107 |
| Moisture -> BMI -> HbA1c |  | 0.107 | 0.256 | **0.027** |
| **BMI** |  |  |  |  |
| BMI -> HbA1c |  |  |  | **0.256** |

**Table S2** Results for females only: significant paths and summarized causal effects for each variable in the path analysis. Dietary variables include energy, protein, carbohydrate, total sugars, total fat and moisture. For indirect effects, intermediate path coefficients are reported under the columns of ρ1, ρ2, and ρ3.

| **Paths** | **Intermediate Path Coefficients** | | | **Total Direct/Indirect Effects** |
| --- | --- | --- | --- | --- |
|  | **ρ1** | **ρ2** | **ρ3** |  |
| **Physical Activity** |  |  |  |  |
| Physical Activity -> Magnesium |  |  |  | 0.230 |
| Physical Activity -> Magnesium -> HbA1c |  | 0.230 | -0.171 | -0.039 |
| Physical Activity -> Magnesium -> BMI -> HbA1c | 0.230 | -0.257 | 0.197 | -0.012 |
| Total effects on HbA1c |  |  |  | **-0.051** |
| **Age** |  |  |  |  |
| Age -> HbA1c |  |  |  | **0.293** |
| **Mexican American** |  |  |  |  |
| MexA -> Phosphorus |  |  |  | 0.244 |
| MexA -> Magnesium |  |  |  | 0.389 |
| MexA -> Magnesium -> HbA1c |  | 0.389 | -0.171 | -0.067 |
| MexA -> Phosphorus -> BMI -> HbA1c | 0.244 | 0.214 | 0.197 | 0.010 |
| MexA -> Magnesium -> BMI -> HbA1c | 0.389 | -0.257 | 0.197 | -0.020 |
| Total effects on HbA1c |  |  |  | **-0.077** |
| **Non-Hispanic Black** |  |  |  |  |
| NHB -> HbA1c |  |  |  | 0.273 |
| NHB -> BMI |  |  |  | 0.382 |
| NHB -> Moisture |  |  |  | -0.338 |
| NHB -> BMI -> HbA1c |  | 0.382 | 0.197 | 0.075 |
| Total effects on HbA1c |  |  |  | **0.348** |
| **Education** |  |  |  |  |
| Education -> Phosphorus |  |  |  | 0.105 |
| Education -> Magnesium |  |  |  | 0.180 |
| Education -> Moisture |  |  |  | 0.108 |
| Education -> Magnesium -> HbA1c |  | 0.180 | -0.171 | -0.031 |
| Total effects on HbA1c |  |  |  | **-0.031** |
| **SES** |  |  |  |  |
| SES -> BMI |  |  |  | -0.096 |
| SES -> BMI -> HbA1c |  | -0.096 | 0.197 | **-0.019** |
| **Phosphorus** |  |  |  |  |
| Phosphorus -> BMI |  |  |  | 0.214 |
| Phosphorus -> BMI -> HbA1c |  | 0.214 | 0.197 | **0.042** |
| **Magnesium** |  |  |  |  |
| Magnesium -> HbA1c |  |  |  | -0.171 |
| Magnesium -> BMI |  |  |  | -0.257 |
| Magnesium -> BMI -> HbA1c |  | -0.257 | 0.197 | -0.050 |
| Total effects on HbA1c |  |  |  | **-0.478** |
| **Moisture** |  |  |  |  |
| Moisture -> BMI |  |  |  | 0.134 |
| Moisture -> BMI -> HbA1c |  | 0.134 | 0.197 | **0.026** |
| **BMI** |  |  |  |  |
| BMI -> HbA1c |  |  |  | **0.197** |

**Table S3** Results for Mexican American stratified by gender, reported are significant paths and summarized causal effects for each variable in the path analysis. Only significant direct effects and indirect effects larger than 0.01 are reported in the table.

| **Paths** | **Estimates** |
| --- | --- |
| **Mexican American Male** |  |
| Age -> HbA1c | **0.385** |
| **Mexican American Female** |  |
| Age -> HbA1c | **0.322** |
| Magnesium -> HbA1c | **-0.307** |
| Magnesium -> BMI | -0.357 |

**Table S4** Results for non-Hispanic black stratified by gender, reported are significant paths and summarized causal effects for each variable in the path analysis. Only significant direct effects and indirect effects larger than 0.01 are reported in the table.

| **Paths** | **Intermediate Path Coefficients** | | **Total Direct/Indirect Effects** |
| --- | --- | --- | --- |
|  | **ρ1** | **ρ2** |  |
| **Non-Hispanic Black Males** | | | |
| No significant paths were found | | | |
| **Non-Hispanic Black Females** | | | |
| **Age** |  |  |  |
| Age -> HbA1c |  |  | **0.340** |
| **Physical Activity** |  |  |  |
| Physical Activity -> Magnesium |  |  | 0.295 |
| Physical Activity -> Magnesium -> HbA1c | 0.295 | -0.246 | **-0.073** |
| **Education** |  |  |  |
| Education -> Magnesium |  |  | 0.212 |
| Education -> Magnesium -> HbA1c | 0.212 | -0.246 | **-0.052** |
| **Energy** |  |  |  |
| Energy -> BMI |  |  | 0.328 |
| Energy -> BMI -> HbA1c | 0.328 | 0.145 | **0.048** |
| **Carbohydrate** |  |  |  |
| Carbohydrate -> BMI |  |  | -0.489 |
| Carbohydrate -> BMI -> HbA1c | -0.489 | 0.145 | **-0.071** |
| **Phosphorus** |  |  |  |
| Phosphorus -> HbA1c |  |  | 0.297 |
| Phosphorus -> BMI |  |  | 0.312 |
| Phosphorus -> BMI -> HbA1c | 0.312 | 0.145 | 0.045 |
| Total effects on HbA1c |  |  | **0.342** |
| **Magnesium** |  |  |  |
| Magnesium -> HbA1c |  |  | **-0.246** |
| **BMI** |  |  |  |
| BMI -> HbA1c |  |  | **0.145** |

**Table S5** Results for non-Hispanic white stratified by gender, reported are significant paths and summarized causal effects for each variable in the path analysis. Only significant direct effects and indirect effects larger than 0.01 are reported in the table.

| **Paths** | **Intermediate Path Coefficients** | | | **Total Direct/Indirect Effects** |
| --- | --- | --- | --- | --- |
|  | **ρ1** | **ρ2** | **ρ3** |  |
| **Non-Hispanic White Male** | | | | |
| **Age** |  |  |  |  |
| Age -> HbA1c |  |  |  | 0.226 |
| Age -> Energy |  |  |  | -0.149 |
| Age -> Carbohydrate |  |  |  | -0.168 |
| Age -> Total_Sugars |  |  |  | -0.167 |
| Age -> Energy -> HbA1c |  | -0.149 | -0.588 | 0.088 |
| Age -> Energy -> BMI -> HbA1c | -0.149 | 0.307 | 0.240 | -0.011 |
| Age -> Carbohydrate -> HbA1c |  | -0.168 | 0.327 | -0.055 |
| Age -> Carbohydrate -> BMI -> HbA1c | -0.168 | -0.399 | 0.240 | 0.016 |
| Total effects on HbA1c |  |  |  | **0.264** |
| **Education** |  |  |  |  |
| Education -> HbA1c |  |  |  | **-0.115** |
| **Energy** |  |  |  |  |
| Energy -> HbA1c |  |  |  | -0.588 |
| Energy -> BMI |  |  |  | 0.307 |
| Energy -> BMI -> HbA1c |  | 0.307 | 0.240 | 0.074 |
| Total effects on HbA1c |  |  |  | **-0.514** |
| **Protein** |  |  |  |  |
| Protein -> HbA1c |  |  |  | **0.145** |
| **Carbohydrate** |  |  |  |  |
| Carbohydrate -> HbA1c |  |  |  | 0.327 |
| Carbohydrate -> BMI |  |  |  | -0.399 |
| Carbohydrate -> BMI -> HbA1c |  | -0.399 | 0.240 | -0.096 |
| Total effects on HbA1c |  |  |  | **0.231** |
| **Total Fat** |  |  |  |  |
| Total Fat -> HbA1c |  |  |  | **0.216** |
| **Thiamin (Vitamin B1)** |  |  |  |  |
| Thiamin VitB1 -> BMI |  |  |  | 0.120 |
| Thiamin VitB1 -> BMI -> HbA1c |  | 0.120 | 0.240 | **0.029** |
| **BMI** |  |  |  |  |
| BMI -> HbA1c |  |  |  | **0.240** |
| **Non-Hispanic White Female** | | | | |
| **Age** |  |  |  |  |
| Age -> HbA1c |  |  |  | **0.273** |
| **Physical Activity** |  |  |  |  |
| Physical Activity -> BMI |  |  |  | -0.356 |
| Physical Activity -> BMI -> HbA1c |  | -0.356 | 0.228 | **-0.081** |
| **Education** |  |  |  |  |
| Education -> VitC |  |  |  | 0.170 |
| Education -> Alcohol |  |  |  | 0.119 |
| **Alcohol** |  |  |  |  |
| Alcohol -> BMI |  |  |  | -0.193 |
| Alcohol -> BMI -> HbA1c |  | -0.193 | 0.228 | **-0.044** |
| **SFA 4:0 (Butanoic)** |  |  |  |  |
| SFA_40_Butanoic -> BMI |  |  |  | -0.136 |
| SFA_40_Butanoic -> BMI -> HbA1c |  | -0.136 | 0.228 | **-0.031** |
| **BMI** |  |  |  |  |
| BMI -> HbA1c |  |  |  | **0.228** |

**Figure S1**: Diagram on creating the study cohort based on the inclusion/exclusion criteria.

NHANES 2013-2014

Complete demographic data

N=10175

NHANES 2011-2012

Complete demographic data

N=9756

NHANES 2011-2014

N=19931

20 ≤ Age < 80

N=10614

Race: non-Hispanic white, non-Hispanic black and Mexican American

N=7826

Complete information in BMI, physical activities and HbA1c

N=3357

Complete information in Dietary

N=2832

Subjects who took only oral hypoglycemic agents

N=2831


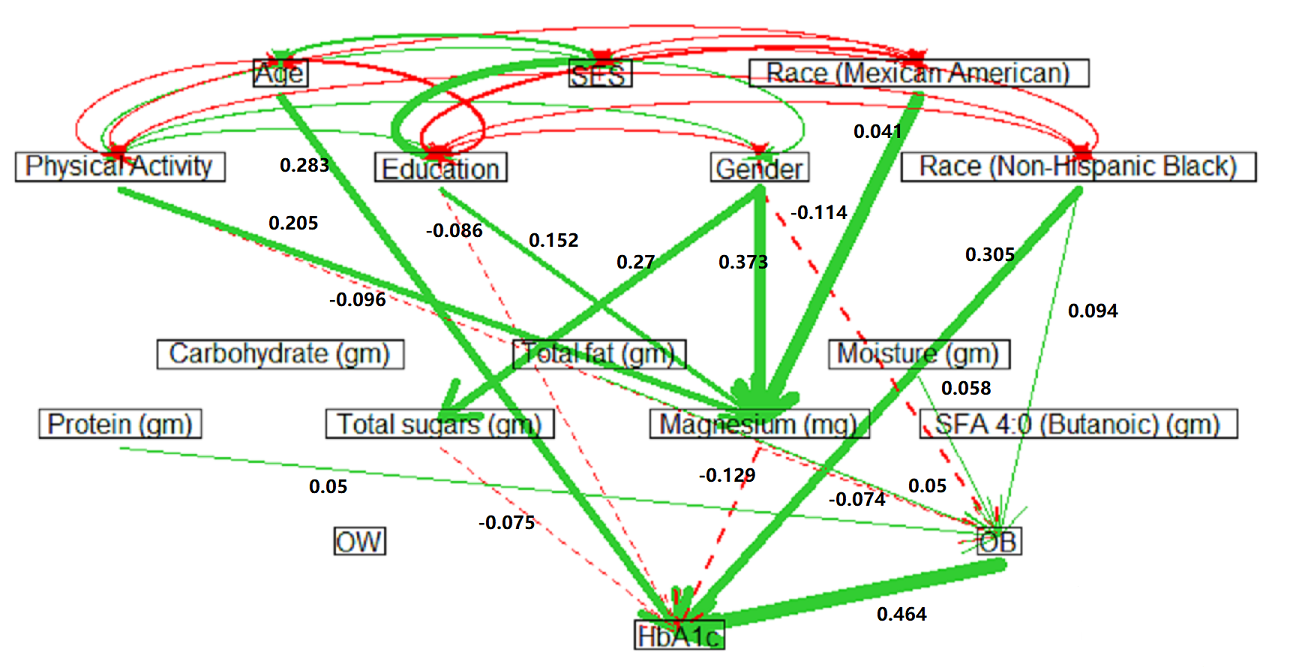


**Figure S2** Path diagram for whole population using three different levels of BMI (normal, overweight (OW), and obese(OB)). Solid (green) paths indicate positive path coefficients, while dashed (red) paths indicate negative path coefficients. The widths of the paths are related to the absolute values of path coefficients, where higher absolute value (i.e., wider path) indicates stronger causality. Only significant paths are shown in the diagram.
